# Factors associated with late recurrence after completion of 5-year adjuvant tamoxifen in estrogen receptor positive breast cancer

**DOI:** 10.1186/s12885-016-2423-x

**Published:** 2016-07-07

**Authors:** Eun-Shin Lee, Wonshik Han, Min Kyoon Kim, Jongjin Kim, Tae-kyung Yoo, Moo Hyun Lee, Kyung Hun Lee, Tae Yong Kim, Hyeong-Gon Moon, Seock-Ah Im, Dong-Young Noh, Eun Sook Lee

**Affiliations:** Department of Surgery, Seoul National University College of Medicine, Seoul, Korea; Department of Surgery, Kangwon National University Hospital, Chuncheon, Korea; Department of Surgery, SMG - SNU Boramae Medical Center, Seoul, Korea; Department of Surgery, Center for Breast Cancer, Research Institute and Hospital, National Cancer Center, Goyang, Korea; Department of Internal Medicine, Seoul National University College of Medicine, Seoul, Korea; Department of Surgery, Seoul National University Hospital, National University College of Medicine, Cancer Research Institute, Seoul National University College of Medicine, 28 Yongon-dong, Chongno-gu, Seoul, 110-744 Korea

**Keywords:** Estrogen receptor (ER)-positive breast cancer, Late recurrence, Extended tamoxifen

## Abstract

**Background:**

Recent large trials have shown the survival benefits of 10-year use of tamoxifen by reducing late recurrence compared with 5-year therapy in estrogen receptor(ER)-positive breast cancer. We tried to identify clinical factors associated with the late recurrence.

**Methods:**

We reviewed our database of ER-positive patients who had received operations between 1996 and 2006 in two institutions. We selected 444 who had completed 5-year tamoxifen and were disease-free up to 10 years after the operation. Patients who had received aromatase inhibitors with any regimens were excluded. As a late recurrence group, 139 patients were identified who had completed 5-year tamoxifen, but had recurrence afterwards. Among them, 61 had local/contralateral breast recurrence and 78 had distant metastasis. The median follow-up was 9.7 years. Clinicopathological factors at the time of initial operation, such as age, menopausal status, progesterone receptor expression, HER2 status, tumor grade and Ki-67, were compared between the disease-free group and the late recurrence group.

**Results:**

In a univariate analysis, tumor size (>2 cm), lymph node metastasis and high histologic grade were significantly associated with late recurrences (*p* < 0.05). In a multivariate analysis, only axillary lymph node metastasis was significant (*p* < 0.001). Late distant metastasis was significantly associated with tumor size and axillary lymph node metastasis (*p* = 0.038, *p* < 0.001,respectively). Late local/contralateral breast recurrence was associated with axillary lymph node metastasis (*p* = 0.042).

**Conclusions:**

Our data showed axillary lymph node metastasis at initial operation was the only risk factor of late recurrence after completion of tamoxifen for 5 years. Our results can be helpful in making decisions to use extended tamoxifen beyond 5 years.

**Electronic supplementary material:**

The online version of this article (doi:10.1186/s12885-016-2423-x) contains supplementary material, which is available to authorized users.

## Background

The treatment of breast cancer has developed remarkably in recent decades, especially in hormone receptor-positive subtype breast cancer [1–7]. Although adjuvant endocrine therapy was highly effective and could reduce recurrence and the mortality of hormone receptor-positive patients, the long-term follow-up data showed that there was a sustained hazard of recurrence even after the completion of 5 years of adjuvant endocrine therapy [2, 6, 8–10]. Therefore, strategies to reduce late recurrence in this subtype of breast cancer have been intensively studied [11–15].

For the past few decades, 5-year use of tamoxifen has been a standard adjuvant endocrine therapy with a large survival gain and minimal adverse effects [8, 16–19]. Studies have shown that tamoxifen therapy has a carryover effect, which results in the reduction in recurrence well after treatment has stopped [9, 17, 20]. Some earlier studies suggested that use of tamoxifen for more than 5 years has few benefits and increases side effects [17, 19, 21]. However, two recent large clinical trials, ATLAS (Adjuvant Tamoxifen: Longer Against Shorter) and aTTom (adjuvant Tamoxifen–To Offer More?) have shown that continuing tamoxifen therapy beyond 5 years reduces recurrence and death from breast cancer over the following years [22, 23]. Accordingly, the new ASCO guidelines recommend a adjuvant hormonal therapy of women who have hormone receptor–positive breast cancer for a duration of up to 10 years rather than 5 years [24]. The next challenge is to determine which patients will benefit from this long-term treatment, because its side effects, such as menopausal symptoms and the risk of endometrial cancer, are considerable [11, 21].

We analyzed the clinicopathological features at the time of surgery of patients who had late recurrence compared with those of patients who were long-term disease-free. We found predictive factors, which will help clinics to select patients who will benefit more from extended adjuvant tamoxifen use for more than 5 years.

## Methods

### Study subjects

**Fig. 1:**
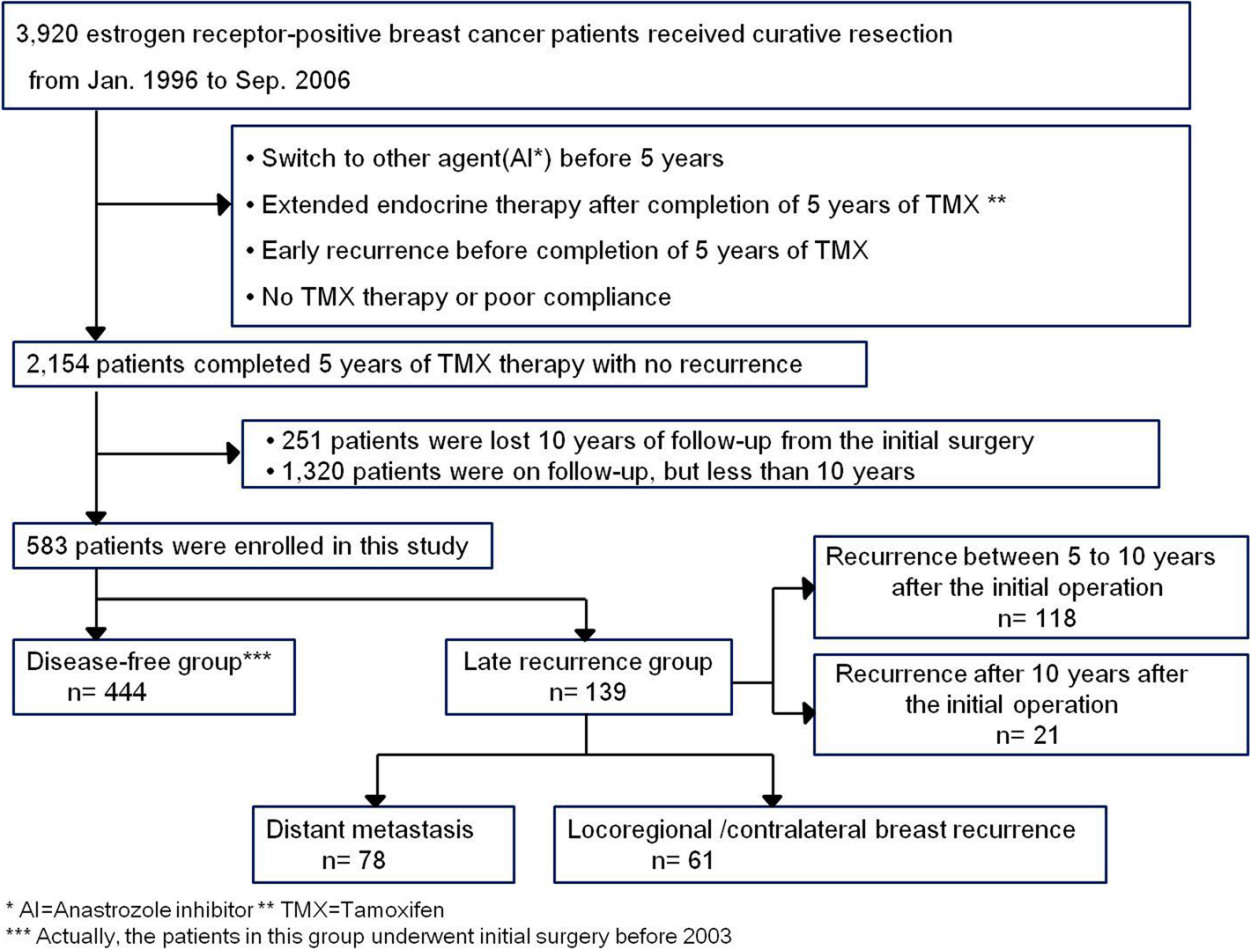
Flowchart of patient selection

We reviewed the data of 3920 patients with estrogen receptor (ER)-positive primary invasive breast cancer who underwent curative surgery in both Seoul National University Hospital and National cancer center from January 1996 to September 2006. We identified 2154 patients who were disease-free when they had finished 5-year adjuvant tamoxifen therapy. Patients who had received aromatase inhibitors at any time during tamoxifen therapy were excluded. Additionally, patients who had received extended endocrine therapy with tamoxifen or aromatase inhibitors for a total duration of more than 5 years were excluded. For the disease-free group, patients who were lost before 10 years of follow-up from the initial surgery were excluded. Late recurrence was defined as any locoregional (in the ipsilateral/contralateral breast, chest wall, or regional lymph nodes including micro-, macro-metastasis and isolated tumor cells in axilla) or distant relapse on the image study or pathologic confirmation occurring after the completion of 5-year adjuvant tamoxifen therapy. Finally, a total of 583 patients were enrolled in this study. A total of 444 were placed in the disease-free group, and 139 were placed in the late recurrence group (Fig. 1). This study was approved by the Institutional Review Board of Seoul National University Hospital (IRB number: 1409-155-616).

### IHC staining and interpretation

This method was previously used in a published study [25]. The samples were immunostained with the following antibodies according to the manufacturers' instructions: Anti-ER (1:100; 1D5; Dako, Glostrup, Denmark), anti-progesterone receptor (PgR) (1:100; 636; Dako), and anti-HER2 (1:200; A0485; Dako). The IHC staining was scored and confirmed by two pathologists who were blinded to the clinical information. Positive ER and PgR expression were defined as nuclear staining in 10 % or more of tumor cells. The HER2 membranous staining was scored on a scale of 0 to 3+ according to the HercepTest protocol. For tissue samples with a HER2 staining score of 2+, additional HER2 FISH testing was performed. HER2 status was considered positive when the IHC score was 3+ or the gene copy ratio of HER2/CEP17 by FISH was 2.0 or higher.

### Statistical analysis

Disease-free survival (DFS) was defined as the length of time after surgery for primary breast cancer to the earliest report of any locoregional or distant recurrence. Pearson’s chi-square test was used to analyze the association between clinicopathological factors and late recurrence. Logistic regression analysis was used for the multivariate analysis of significant variables in the univariate chi-square tests, such as age, menopausal status, tumor size (e.g., ≤2 cm or >2 cm), metastatic axillary lymph nodes (positive or negative), tumor grade (Nottingham Histologic Score 1, 2, or 3), human epidermal growth factor receptor 2 (HER2) status (positive or negative), progesterone receptor expression (positive or negative), and Ki67 level (high (≥10 % in SNUH, ≥14 % in NCC) or low (<10 % in SNUH, <14 % in NCC)) [26]. Kaplan–Meier plots were used to show the survival results and comparison between the groups. All statistical analyses were performed using SPSS Version 19.0 software. All *p* values were two-sided, and *p* < 0.05 was considered significant.

## Results

Table 1 summarizes the clinicopathologic characteristics of this study population. The mean age of patients was 45.5 years old. The mean follow-up period was 10.6 years in disease-free patients, and the mean disease-free time was 8.0 years (ranging from 5.1 to 14.3 years) in the late recurrence group. A total of 444 patients of 583 (76.2 %) had disease-free status by the end of the follow-up date, at least 10 years after diagnosis. A total of 139 patients (23.8 %) experienced local or distant recurrences after completion of adjuvant tamoxifen therapy for 5 years. Recurrences occurred between 5 to 10 years after the operation in 118 (84.9 %) women and after 10 years in 21(15.1 %) patients. A total of 61 patients experienced locoregional or contralateral breast recurrence, and 78 experienced distant metastasis (Table 2).

**Table 1:** Clinicopathologic characteristics of study subjects

Factors	*N* = 583
Mean age	45.5 (22 ~ 73)
Menopausal status	
Pre-menopause	411 (70.5 %)
Post-menopause	148 (25.4)
Unknown	24 (4.1 %)
Tumor size	
≤2 cm	333 (57.1 %)
>2 cm	250 (42.9 %)
Axillary nodal status	
Node positive (micro- & macro-metastasis/isolated tumor cell)	223 (38.3 %)
Node negative	360 (61.7 %)
AJCC stage	
I	247 (42.4 %)
II	272 (46.7 %)
III	64 (10.9 %)
Histologic type	
Ductal carcinoma	522 (89.5 %)
Lobular carcinoma	17 (2.9 %)
Others	44 (7.6 %)
Progesterone receptor	
Positive	383 (65.7 %)
Negative	198 (34.0 %)
Unknown	2 (0.3 %)
HER2	
Positive	86 (14.7 %)
Negative	328 (56.3 %)
Unknown	169 (29 %)
Nottingham Histologic Score	
1	78 (13.4 %)
2	314 (53.9 %)
3	122 (20.9 %)
Unknown	69 (11.8 %)
Ki-67	
High (≥10 %, or ≥14 %)^a^	120 (20.6 %)
Low (<10 %, or <14 %)^a^	425 (72.9 %)
Unkonwn	38 (6.5 %)
Surgery-Breast	
Mastectomy	308 (52.8 %)
Breast conservation^b^	275 (47.2 %)
Surgery-Axilla	
SLNBx^c^ Only	19 (3.3 %)
ALND^d^	557 (95.5 %)
Others	7 (1.2 %)
Adjuvant treatment	
Radiotherapy	197 (33.8 %)
Chemotherapy	307 (52.7 %)

^a^The cutoff values of Ki-67 are 14 % in NCC and 10 % in SNUH [26]

^b^All patients obtained clear resection margin

^c^ *SLNBx*., Sentinel lymph node biopsy

^d^ *ALND*, axillary lymph node dissection

**Table 2:** Recurrence type in 139 patients of late recurrence

Site of recurrence	*N*
Contralateral breast	34
Local recurrence	27
Chest wall	9
Remnant breast	16
Axillary lymph node	2
Distant metastasis	78
Visceral metastasis (≥1 organ)^a^	48
Non-visceral metastasis	30
Bone metastasis only	26
Multiple metastasis in non-visceral oragan^b^	4

^a^Lung, liver, brain metastasis with or without bone metastasis

^b^Bone metastasis with lymph node metastasis such as internal mammary LN or subclavian LN

**Fig. 2:**
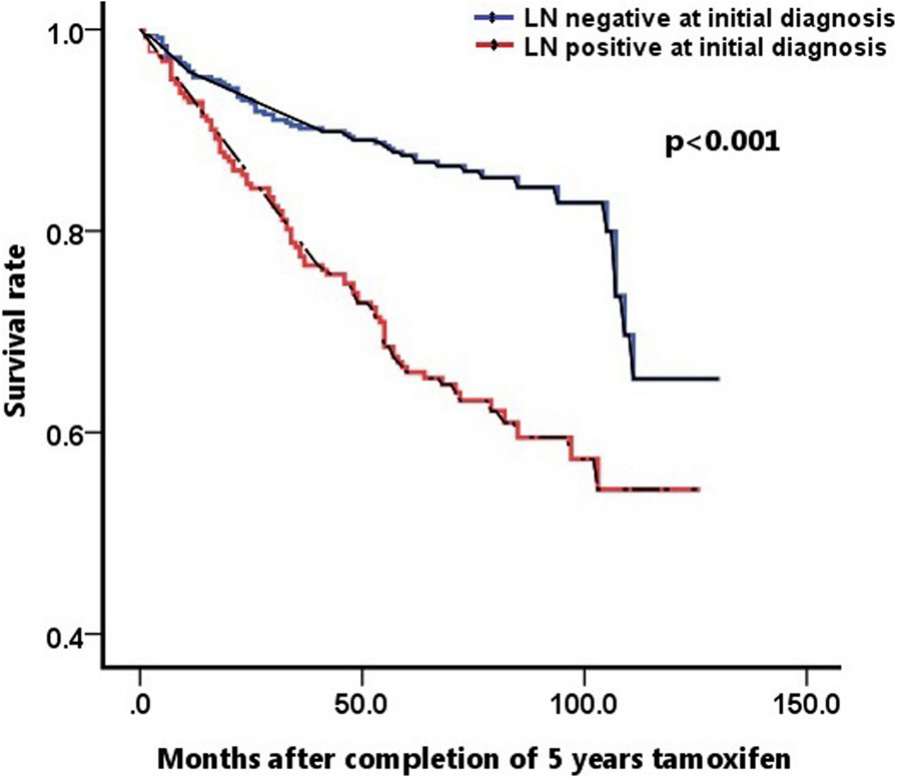
Kaplan–Meier analysis of disease-free survival according to lymph node status at initial operation

In the univariate analysis, large tumor size (>2 cm), positive axillary lymph node metastasis, and high histologic grade were significantly related to late recurrence (*p* = 0.002, *p* < 0.001, and *p* = 0.018, respectively). In the multivariate logistic regression analysis, only axillary lymph node metastasis at the time of initial operation was significantly associated with late recurrence compared with the disease-free group (*p* < 0.001) (Table 3). In the subgroup analysis, distant metastasis in the late recurrence group was significantly associated with axillary lymph node metastasis and large tumor size (*p* < 0.001, *p* = 0.038, respectively), and local recurrence or contralateral breast recurrence was associated with only axillary lymph node metastasis (*p* = 0.042) (Table 4). Figure 2 shows Kaplan–Meier curves for DFS according to lymph node status. Patients who were lymph node-positive at the time of initial operation had significantly worse survival rates after the completion of 5-year adjuvant tamoxifen therapy (log rank *p*-value <0.001).

**Table 3:** Univariate (chi-square) and multivariate (logistic regression) analysis for clinicopathological features associated with late recurrence after completion of 5 years tamoxifen

Variables	Univariate analysis	Multivariate analysis		
	*P* value	Odds ratio	95 % CI	*P* value
Age (<50 *vs* ≥ 50 Years)	0.098	0.981	0.954–1.008	0.168
Menopausal status (pre-*vs*-post-)	0.409			
Tumor size (>2 cm *vs* ≤2 cm)	0.002	1.297	0.762–2.207	0.338
ALN^a^ metastasis (yes *vs* no)	<0.001	2.731	1.615–4.618	<0.001
PgR^b^ (negative *vs* posituve)	0.420			
HER2 (positive *vs* negative)	0.051			
Histologic grade (Gr III *vs* Gr I or II)	0.018	1.396	0.786–2.479	0.256
Ki-67 (high *vs* low)^c^	0.480			
Radiotherapy (yes *vs* no)	0.134			
Chemotheraphy (yes *vs* no)	0.434			

^a^ALN axillary lymph node

^b^PgR progesterone

^c^The cutoff values of Ki-67 are 14 % in NCC and 10 % in SNUH [26]

**Table 4:** Subgroup analysis for associated factors with local or distant metastasis respectively

Variables	Local + contralateral breast recurrence		Distant metastasis	
	*N* = 61		*N* = 78	
	Odds Ratio (95 % CI)	*P* value	Odds Ratio (95 % CI)	*P* value
Age(<50 *vs* ≥50)	0.970 (0.938 ~ 1.004)	0.084	0.984 (0.955 ~ 1.013)	0.282
Tumor size (>2 cm *vs* ≤ 2 cm)	0.834 (0.467 ~ 1.489)	0.539	1.767 (1.033 ~ 3.023)	0.038
ALN^a^ metastasis (Yes *vs* No)	1.817 (1.022 ~ 3.229)	0.042	4.274 (2.470-7.395)	<0.001

^a^ *ALN* axillary lymph node

## Discussion

Although there have been great advances in the survival outcomes of patients with ER-positive breast cancer, many patients still experience late recurrence [1, 2]. The use of extended endocrine therapy for more than 5 years in order to reduce late recurrence is controversial [11, 27]. Due to the publication of positive results from large randomized trials that have shown the benefits of 10-year tamoxifen therapy, new ASCO guidelines recommend a total of 10 years of adjuvant hormonal therapy for all ER-positive patients [22, 24]. However, it is not clear whether all ER-positive breast cancer patients should be offered extended endocrine treatment. The important question is which patients would be at a higher risk of recurrence after 5 years of endocrine therapy and which would benefit from extended therapy.

We demonstrated an association between axillary lymph node metastasis at the time of initial operation and late recurrence in patients who completed 5-year tamoxifen therapy. This result can be helpful for clinics when considering the extended use of tamoxifen beyond 5 years. Doctors might be able to strongly recommend this therapy when the patient is lymph node-positive at the time of initial surgery. Several previous reports have shown similar results [12, 28, 29]. A meta-analysis conducted by Al-Mubarak et al. on extended adjuvant endocrine therapy for early breast cancer showed that the apparent benefits were observed only among patients with lymph node-positive disease, and the absolute risk reduction during 10 years of follow-up was almost doubled in lymph node-positive patients [30].

In this study, it is notable that tumor biological factors known as predictors of early recurrence, such as Ki67 or HER2, were not predictors of late recurrence. This suggests the necessity of developing a new biomarker with good prediction accuracy for late recurrence in ER-positive disease. Recently, multigene assays have shown promising results in the prediction of late recurrence [29, 31–34]. Sgroi et al. compared the prognostic efficacies of the breast-cancer index (BCI) assay, 21-gene recurrence score (Oncotype DX), and an immunohistochemical prognostic model (IHC4). As a result, BCI assay provided significant prognostic information for both early and late distant recurrence [31]. Another multigene-based assay, the EndoPredict test combined with nodal status and tumor size (EPclin), reliably identified a subgroup of patients who showed excellent long-term prognoses after 5 years of endocrine therapy [32]. In a recent large combined analysis of TranATAC and ABCSG8 studies, the risk of recurrence(ROR) score of the PAM50 added clinically meaningful prognostic information to the Clinical Treatment Score in all patients and subgroups in the late follow-up period. They suggested that the ROR score could be helpful for separating into risk groups patients who could be spared or potentially benefit from extended hormonal therapy beyond 5 years of treatment [35].

In our study, the small number of late recurrence events was a weakness. This limitation is due to the fact that the study was performed in just two institution. It was also because we excluded patients whose follow-up duration was not sufficient and who took aromatase inhibitors in any sequence with adjuvant tamoxifen. Another limitation of our study was considerable missing data on HER2 status. Before the adjuvant trastuzumab era, HER2 status was determined by IHC alone. We regarded HER2 as unknown when the HER2 IHC score was 2+ and HER2 FISH data was not available for the patient. Moreover, the absence of information on the degree of ER expression known to be directly associated with the benefits of tamoxifen [8] was another limitation of our study.

## Conclusions

Our data showed that axillary lymph node metastasis at the time of initial operation was significantly associated with late recurrence after completion of 5-year tamoxifen therapy. This result would be useful for making decisions regarding using extended tamoxifen therapy for more than 5 years and when multi-gene assays are not available.

## Abbreviations

AI, anastrozole inhibitor; ALN, axillary lymph node; ALND, axillary lymph node dissection; BCI, breast-cancer index; DFS, disease-free survival; ER, estrogen receptor; HER2, human epidermal growth factor receptor 2; IHC, immunohistochemistry; LN, lymph node; PgR, progesterone receptor; ROR, risk of recurrence; SLNBx., sentinel lymph node biopsy; TMX, tamoxifen

## Additional file

Additional file 1: This file includes raw-data of 583 patients’ characteristics and survival. (XLSX 40 kb)
